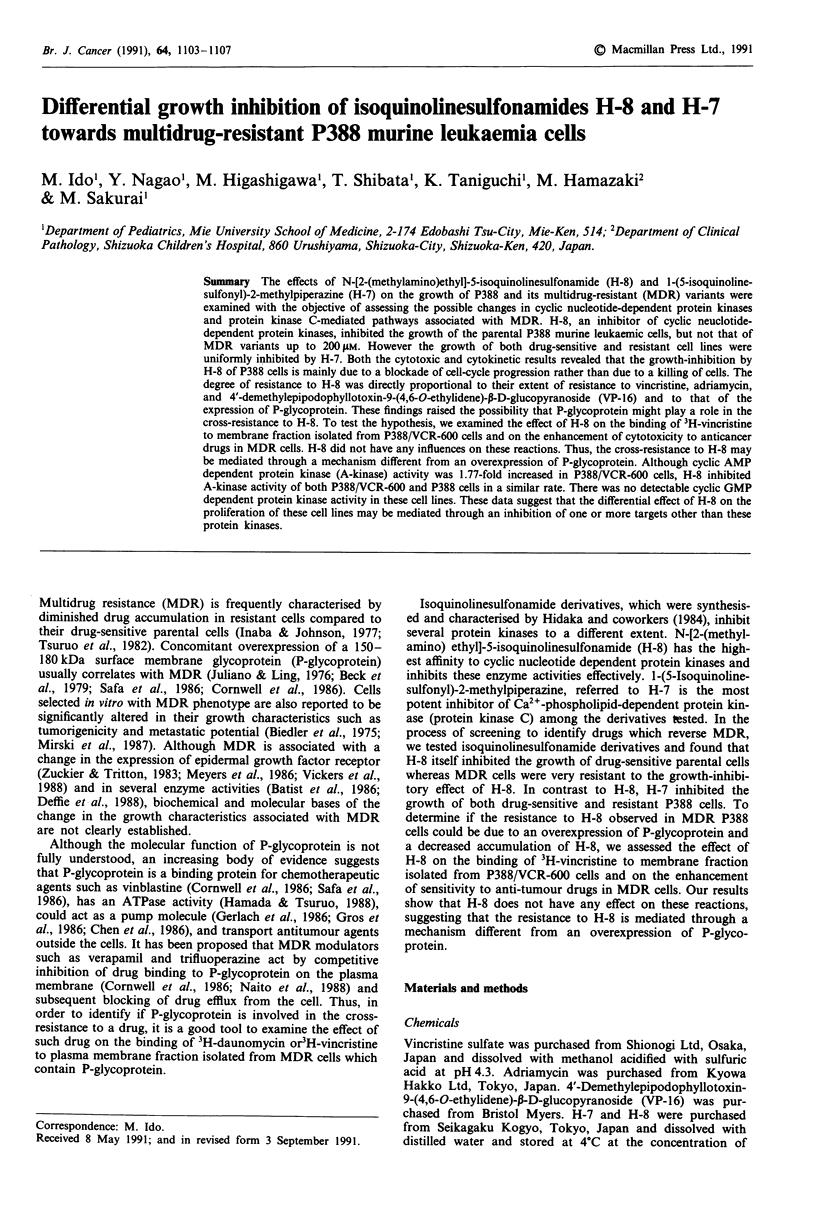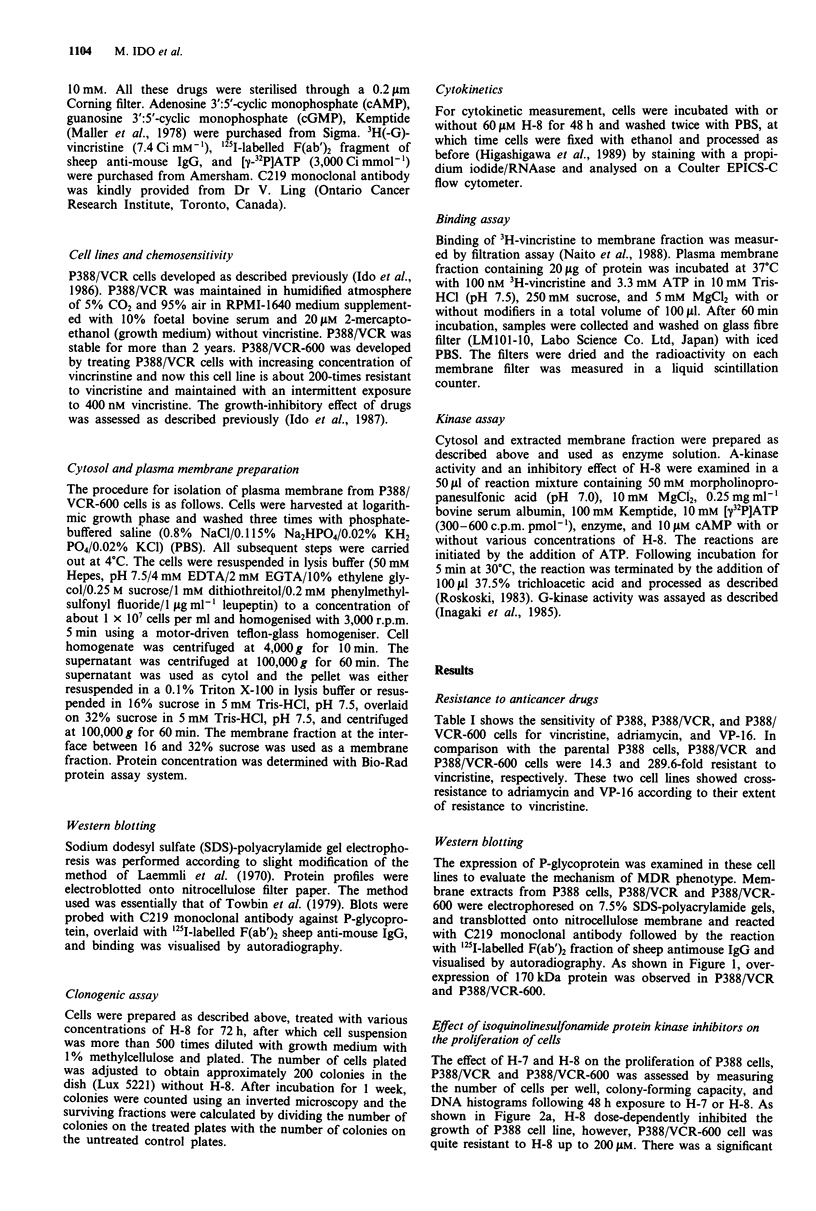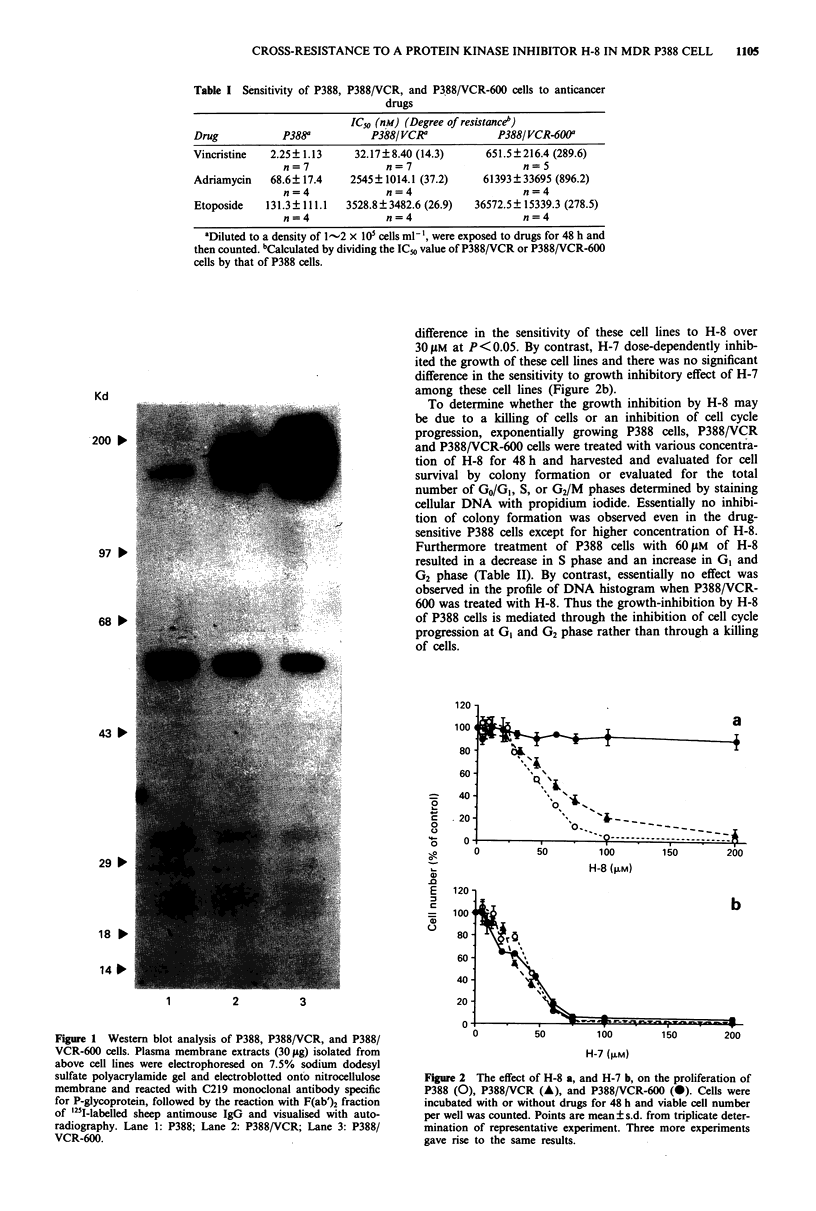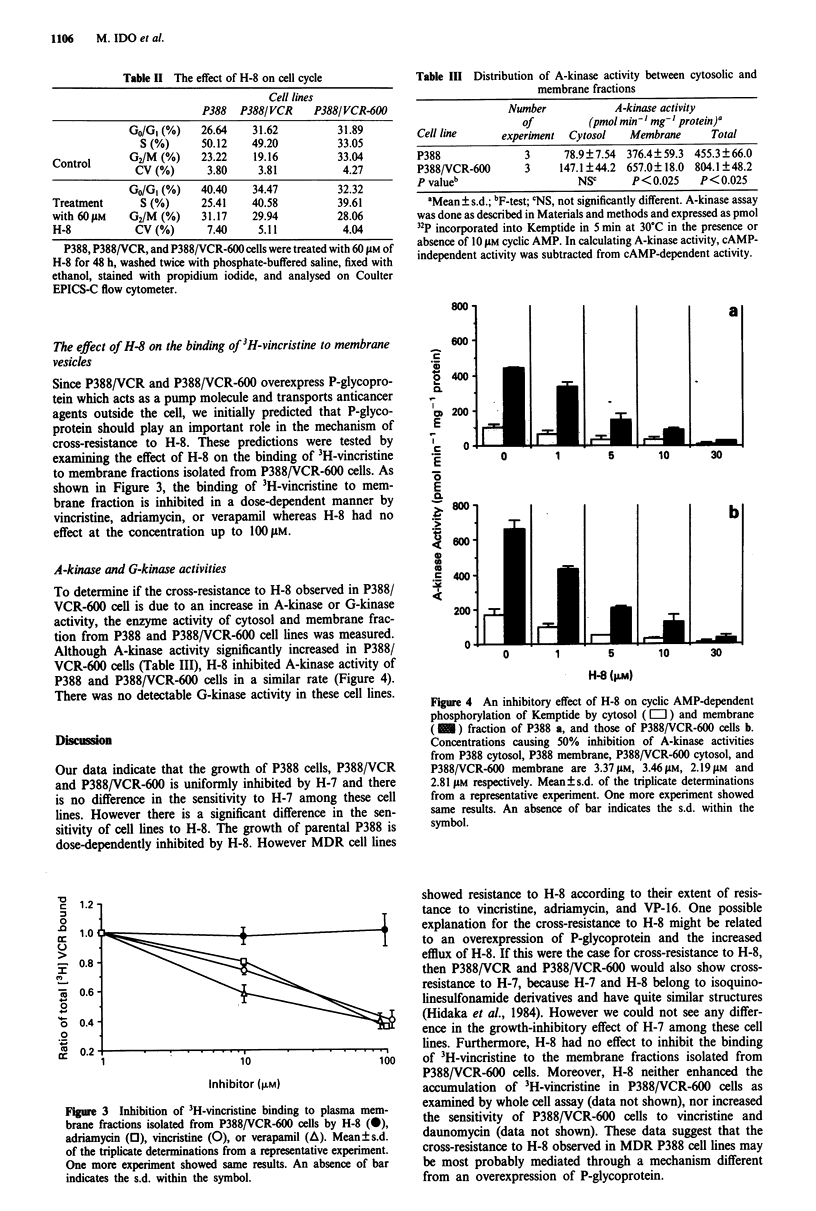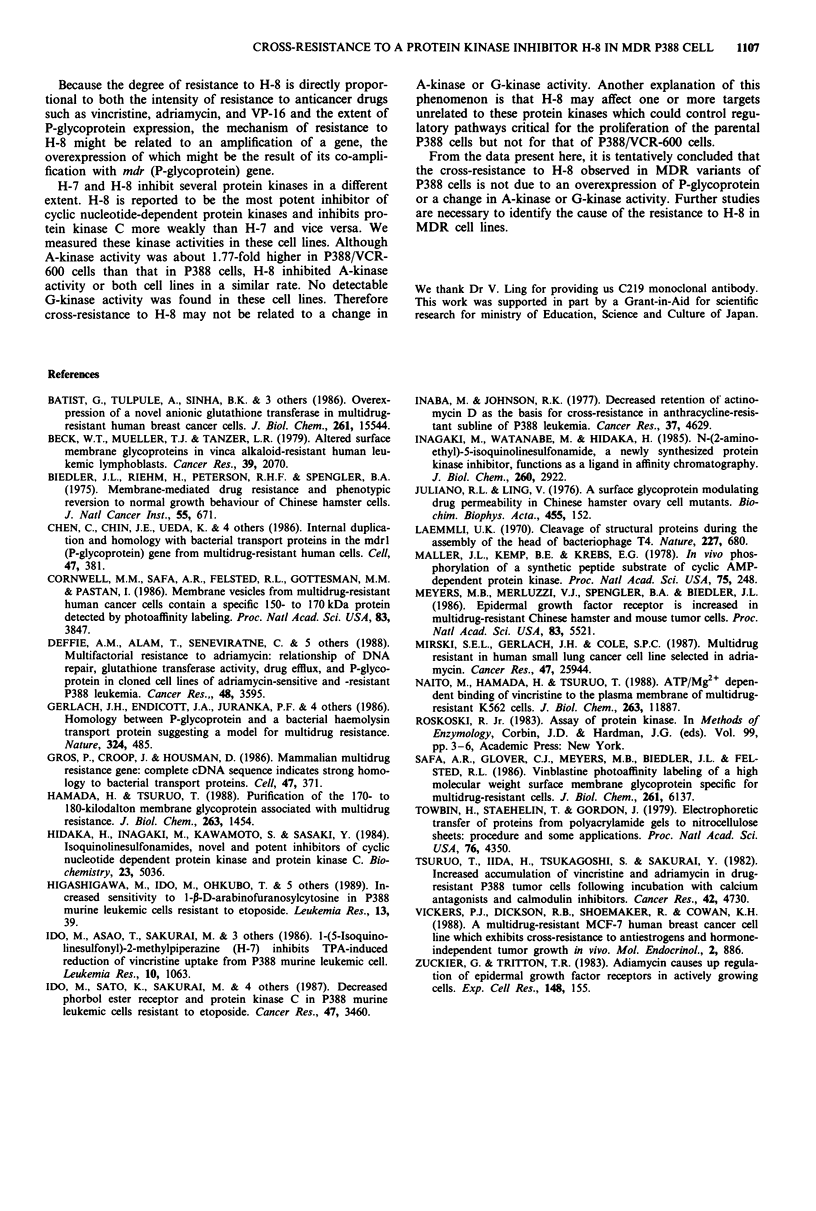# Differential growth inhibition of isoquinolinesulfonamides H-8 and H-7 towards multidrug-resistant P388 murine leukaemia cells.

**DOI:** 10.1038/bjc.1991.472

**Published:** 1991-12

**Authors:** M. Ido, Y. Nagao, M. Higashigawa, T. Shibata, K. Taniguchi, M. Hamazaki, M. Sakurai

**Affiliations:** Department of Pediatrics, Mie University School of Medicine, Japan.

## Abstract

**Images:**


Br. J. Cancer (1991), 64, 1103-1107 © Macmillan Press Ltd., 1991

### Differential growth inhibition of isoquinolinesulfonamides H-8 and H-7 towards multidrug-resistant P388 murine leukaemia cells

M. Ido[1], Y. Nagao[1], M. Higashigawa[1], T. Shibata[1], K. Taniguchi[1], M. Hamazaki[2] & M. Sakurai[1]

[1]*Department of Pediatrics, Mie University School of Medicine, 2-174 Edobashi Tsu-City, Mie-Ken, 514;* [2]*Department of Clinical Pathology, Shizuoka Children's Hospital, 860 Urushiyama, Shizuoka-City, Shizuoka-Ken, 420, Japan.*

**Summary** The effects of N-[2-(methylamino)ethyl]-5-isoquinolinesulfonamide (H-8) and 1-(5-isoquinolinesulfonyl)-2-methylpiperazine (H-7) on the growth of P388 and its multidrug-resistant (MDR) variants were examined with the objective of assessing the possible changes in cyclic nucleotide-dependent protein kinases and protein kinase C-mediated pathways associated with MDR. H-8, an inhibitor of cyclic neuclotide-dependent protein kinases, inhibited the growth of the parental P388 murine leukaemic cells, but not that of MDR variants up to 200 μM. However the growth of both drug-sensitive and resistant cell lines were uniformly inhibited by H-7. Both the cytotoxic and cytokinetic results revealed that the growth-inhibition by H-8 of P388 cells is mainly due to a blockade of cell-cycle progression rather than due to a killing of cells. The degree of resistance to H-8 was directly proportional to their extent of resistance to vincristine, adriamycin, and 4'-demethylepipodophyllotoxin-9-(4,6-*O*-ethylidene)-β-D-glucopyranoside (VP-16) and to that of the expression of P-glycoprotein. These findings raised the possibility that P-glycoprotein might play a role in the cross-resistance to H-8. To test the hypothesis, we examined the effect of H-8 on the binding of $^3$H-vincristine to membrane fraction isolated from P388/VCR-600 cells and on the enhancement of cytotoxicity to anticancer drugs in MDR cells. H-8 did not have any influences on these reactions. Thus, the cross-resistance to H-8 may be mediated through a mechanism different from an overexpression of P-glycoprotein. Although cyclic AMP dependent protein kinase (A-kinase) activity was 1.77-fold increased in P388/VCR-600 cells, H-8 inhibited A-kinase activity of both P388/VCR-600 and P388 cells in a similar rate. There was no detectable cyclic GMP dependent protein kinase activity in these cell lines. These data suggest that the differential effect of H-8 on the proliferation of these cell lines may be mediated through an inhibition of one or more targets other than these protein kinases.

Multidrug resistance (MDR) is frequently characterised by diminished drug accumulation in resistant cells compared to their drug-sensitive parental cells (Inaba & Johnson, 1977; Tsuruo *et al.*, 1982). Concomitant overexpression of a 150–180 kDa surface membrane glycoprotein (P-glycoprotein) usually correlates with MDR (Juliano & Ling, 1976; Beck *et al.*, 1979; Safa *et al.*, 1986; Cornwell *et al.*, 1986). Cells selected *in vitro* with MDR phenotype are also reported to be significantly altered in their growth characteristics such as tumorigenicity and metastatic potential (Biedler *et al.*, 1975; Mirski *et al.*, 1987). Although MDR is associated with a change in the expression of epidermal growth factor receptor (Zuckier & Tritton, 1983; Meyers *et al.*, 1986; Vickers *et al.*, 1988) and in several enzyme activities (Batist *et al.*, 1986; Deffie *et al.*, 1988), biochemical and molecular bases of the change in the growth characteristics associated with MDR are not clearly established.

Although the molecular function of P-glycoprotein is not fully understood, an increasing body of evidence suggests that P-glycoprotein is a binding protein for chemotherapeutic agents such as vinblastine (Cornwell *et al.*, 1986; Safa *et al.*, 1986), has an ATPase activity (Hamada & Tsuruo, 1988), could act as a pump molecule (Gerlach *et al.*, 1986; Gros *et al.*, 1986; Chen *et al.*, 1986), and transport antitumour agents outside the cells. It has been proposed that MDR modulators such as verapamil and trifluoperazine act by competitive inhibition of drug binding to P-glycoprotein on the plasma membrane (Cornwell *et al.*, 1986; Naito *et al.*, 1988) and subsequent blocking of drug efflux from the cell. Thus, in order to identify if P-glycoprotein is involved in the cross-resistance to a drug, it is a good tool to examine the effect of such drug on the binding of $^3$H-daunomycin or $^3$H-vincristine to plasma membrane fraction isolated from MDR cells which contain P-glycoprotein.

Correspondence: M. Ido.
Received 8 May 1991; and in revised form 3 September 1991.

Isoquinolinesulfonamide derivatives, which were synthesised and characterised by Hidaka and coworkers (1984), inhibit several protein kinases to a different extent. N-[2-(methylamino) ethyl]-5-isoquinolinesulfonamide (H-8) has the highest affinity to cyclic nucleotide dependent protein kinases and inhibits these enzyme activities effectively. 1-(5-Isoquinolinesulfonyl)-2-methylpiperazine, referred to H-7 is the most potent inhibitor of $Ca^{2+}$-phospholipid-dependent protein kinase (protein kinase C) among the derivatives tested. In the process of screening to identify drugs which reverse MDR, we tested isoquinolinesulfonamide derivatives and found that H-8 itself inhibited the growth of drug-sensitive parental cells whereas MDR cells were very resistant to the growth-inhibitory effect of H-8. In contrast to H-8, H-7 inhibited the growth of both drug-sensitive and resistant P388 cells. To determine if the resistance to H-8 observed in MDR P388 cells could be due to an overexpression of P-glycoprotein and a decreased accumulation of H-8, we assessed the effect of H-8 on the binding of $^3$H-vincristine to membrane fraction isolated from P388/VCR-600 cells and on the enhancement of sensitivity to anti-tumour drugs in MDR cells. Our results show that H-8 does not have any effect on these reactions, suggesting that the resistance to H-8 is mediated through a mechanism different from an overexpression of P-glycoprotein.

### Materials and methods

### *Chemicals*

Vincristine sulfate was purchased from Shionogi Ltd, Osaka, Japan and dissolved with methanol acidified with sulfuric acid at pH 4.3. Adriamycin was purchased from Kyowa Hakko Ltd, Tokyo, Japan. 4'-Demethylepipodophyllotoxin-9-(4,6-*O*-ethylidene)-β-D-glucopyranoside (VP-16) was purchased from Bristol Myers. H-7 and H-8 were purchased from Seikagaku Kogyo, Tokyo, Japan and dissolved with distilled water and stored at 4°C at the concentration of

10 mM. All these drugs were sterilised through a 0.2 μm Corning filter. Adenosine 3':5'-cyclic monophosphate (cAMP), guanosine 3':5'-cyclic monophosphate (cGMP), Kemptide (Maller *et al.*, 1978) were purchased from Sigma. $^3$H(-G)-vincristine (7.4 Ci mM$^{-1}$), $^{125}$I-labelled F(ab')$_2$ fragment of sheep anti-mouse IgG, and [γ-$^{32}$P]ATP (3,000 Ci mmol$^{-1}$) were purchased from Amersham. C219 monoclonal antibody was kindly provided from Dr V. Ling (Ontario Cancer Research Institute, Toronto, Canada).

### *Cell lines and chemosensitivity*

P388/VCR cells developed as described previously (Ido *et al.*, 1986). P388/VCR was maintained in humidified atmosphere of 5% $CO_2$ and 95% air in RPMI-1640 medium supplemented with 10% foetal bovine serum and 20 μM 2-mercaptoethanol (growth medium) without vincristine. P388/VCR was stable for more than 2 years. P388/VCR-600 was developed by treating P388/VCR cells with increasing concentration of vincrinstine and now this cell line is about 200-times resistant to vincristine and maintained with an intermittent exposure to 400 nM vincristine. The growth-inhibitory effect of drugs was assessed as described previously (Ido *et al.*, 1987).

### *Cytosol and plasma membrane preparation*

The procedure for isolation of plasma membrane from P388/VCR-600 cells is as follows. Cells were harvested at logarithmic growth phase and washed three times with phosphate-buffered saline (0.8% NaCl/0.115% $Na_2HPO_4$/0.02% $KH_2PO_4$/0.02% KCl) (PBS). All subsequent steps were carried out at 4°C. The cells were resuspended in lysis buffer (50 mM Hepes, pH 7.5/4 mM EDTA/2 mM EGTA/10% ethylene glycol/0.25 M sucrose/1 mM dithiothreitol/0.2 mM phenylmethylsulfonyl fluoride/1 μg ml$^{-1}$ leupeptin) to a concentration of about 1 × 10$^7$ cells per ml and homogenised with 3,000 r.p.m. 5 min using a motor-driven teflon-glass homogeniser. Cell homogenate was centrifuged at 4,000 *g* for 10 min. The supernatant was centrifuged at 100,000 *g* for 60 min. The supernatant was used as cytol and the pellet was either resuspended in a 0.1% Triton X-100 in lysis buffer or resuspended in 16% sucrose in 5 mM Tris-HCl, pH 7.5, overlaid on 32% sucrose in 5 mM Tris-HCl, pH 7.5, and centrifuged at 100,000 *g* for 60 min. The membrane fraction at the interface between 16 and 32% sucrose was used as a membrane fraction. Protein concentration was determined with Bio-Rad protein assay system.

### *Western blotting*

Sodium dodesyl sulfate (SDS)-polyacrylamide gel electrophoresis was performed according to slight modification of the method of Laemmli *et al.* (1970). Protein profiles were electroblotted onto nitrocellulose filter paper. The method used was essentially that of Towbin *et al.* (1979). Blots were probed with C219 monoclonal antibody against P-glycoprotein, overlaid with $^{125}$I-labelled F(ab')$_2$ sheep anti-mouse IgG, and binding was visualised by autoradiography.

### *Clonogenic assay*

Cells were prepared as described above, treated with various concentrations of H-8 for 72 h, after which cell suspension was more than 500 times diluted with growth medium with 1% methylcellulose and plated. The number of cells plated was adjusted to obtain approximately 200 colonies in the dish (Lux 5221) without H-8. After incubation for 1 week, colonies were counted using an inverted microscopy and the surviving fractions were calculated by dividing the number of colonies on the treated plates with the number of colonies on the untreated control plates.

### *Cytokinetics*

For cytokinetic measurement, cells were incubated with or without 60 μM H-8 for 48 h and washed twice with PBS, at which time cells were fixed with ethanol and processed as before (Higashigawa *et al.*, 1989) by staining with a propidium iodide/RNAase and analysed on a Coulter EPICS-C flow cytometer.

### *Binding assay*

Binding of $^3$H-vincristine to membrane fraction was measured by filtration assay (Naito *et al.*, 1988). Plasma membrane fraction containing 20 μg of protein was incubated at 37°C with 100 nM $^3$H-vincristine and 3.3 mM ATP in 10 mM Tris-HCl (pH 7.5), 250 mM sucrose, and 5 mM $MgCl_2$ with or without modifiers in a total volume of 100 μl. After 60 min incubation, samples were collected and washed on glass fibre filter (LM101-10, Labo Science Co. Ltd, Japan) with iced PBS. The filters were dried and the radioactivity on each membrane filter was measured in a liquid scintillation counter.

### *Kinase assay*

Cytosol and extracted membrane fraction were prepared as described above and used as enzyme solution. A-kinase activity and an inhibitory effect of H-8 were examined in a 50 μl of reaction mixture containing 50 mM morpholinopropanesulfonic acid (pH 7.0), 10 mM $MgCl_2$, 0.25 mg ml$^{-1}$ bovine serum albumin, 100 mM Kemptide, 10 mM [γ$^{32}$P]ATP (300–600 c.p.m. pmol$^{-1}$), enzyme, and 10 μM cAMP with or without various concentrations of H-8. The reactions are initiated by the addition of ATP. Following incubation for 5 min at 30°C, the reaction was terminated by the addition of 100 μl 37.5% trichloacetic acid and processed as described (Roskoski, 1983). G-kinase activity was assayed as described (Inagaki *et al.*, 1985).

### Results

### *Resistance to anticancer drugs*

Table I shows the sensitivity of P388, P388/VCR, and P388/VCR-600 cells for vincristine, adriamycin, and VP-16. In comparison with the parental P388 cells, P388/VCR and P388/VCR-600 cells were 14.3 and 289.6-fold resistant to vincristine, respectively. These two cell lines showed cross-resistance to adriamycin and VP-16 according to their extent of resistance to vincristine.

### *Western blotting*

The expression of P-glycoprotein was examined in these cell lines to evaluate the mechanism of MDR phenotype. Membrane extracts from P388 cells, P388/VCR and P388/VCR-600 were electrophoresed on 7.5% SDS-polyacrylamide gels, and transblotted onto nitrocellulose membrane and reacted with C219 monoclonal antibody followed by the reaction with $^{125}$I-labelled F(ab')$_2$ fraction of sheep antimouse IgG and visualised by autoradiography. As shown in Figure 1, over-expression of 170 kDa protein was observed in P388/VCR and P388/VCR-600.

### *Effect of isoquinolinesulfonamide protein kinase inhibitors on the proliferation of cells*

The effect of H-7 and H-8 on the proliferation of P388 cells, P388/VCR and P388/VCR-600 was assessed by measuring the number of cells per well, colony-forming capacity, and DNA histograms following 48 h exposure to H-7 or H-8. As shown in Figure 2a, H-8 dose-dependently inhibited the growth of P388 cell line, however, P388/VCR-600 cell was quite resistant to H-8 up to 200 μM. There was a significant

**Table I** Sensitivity of P388, P388/VCR, and P388/VCR-600 cells to anticancer drugs

| Drug | $IC_{50}$ (nM) (Degree of resistance[b]) P388[a] | P388/VCR[a] | P388/VCR-600[a] |
|---|---|---|---|
| Vincristine | 2.25±1.13 $n=7$ | 32.17±8.40 (14.3) $n=7$ | 651.5±216.4 (289.6) $n=5$ |
| Adriamycin | 68.6±17.4 $n=4$ | 2545±1014.1 (37.2) $n=4$ | 61393±33695 (896.2) $n=4$ |
| Etoposide | 131.3±111.1 $n=4$ | 3528.8±3482.6 (26.9) $n=4$ | 36572.5±15339.3 (278.5) $n=4$ |

[a]Diluted to a density of 1~2 × $10^5$ cells $ml^{-1}$, were exposed to drugs for 48 h and then counted. [b]Calculated by dividing the $IC_{50}$ value of P388/VCR or P388/VCR-600 cells by that of P388 cells.

**Figure 1** Western blot analysis of P388, P388/VCR, and P388/VCR-600 cells. Plasma membrane extracts (30 μg) isolated from above cell lines were electrophoresed on 7.5% sodium dodecyl sulfate polyacrylamide gel and electroblotted onto nitrocellulose membrane and reacted with C219 monoclonal antibody specific for P-glycoprotein, followed by the reaction with $F(ab')_2$ fraction of $^{125}I$-labelled sheep antimouse IgG and visualised with autoradiography. Lane 1: P388; Lane 2: P388/VCR; Lane 3: P388/VCR-600.

difference in the sensitivity of these cell lines to H-8 over 30 μM at $P<0.05$. By contrast, H-7 dose-dependently inhibited the growth of these cell lines and there was no significant difference in the sensitivity to growth inhibitory effect of H-7 among these cell lines (Figure 2b).

To determine whether the growth inhibition by H-8 may be due to a killing of cells or an inhibition of cell cycle progression, exponentially growing P388 cells, P388/VCR and P388/VCR-600 cells were treated with various concentration of H-8 for 48 h and harvested and evaluated for cell survival by colony formation or evaluated for the total number of $G_0/G_1$, S, or $G_2/M$ phases determined by staining cellular DNA with propidium iodide. Essentially no inhibition of colony formation was observed even in the drug-sensitive P388 cells except for higher concentration of H-8. Furthermore treatment of P388 cells with 60 μM of H-8 resulted in a decrease in S phase and an increase in $G_1$ and $G_2$ phase (Table II). By contrast, essentially no effect was observed in the profile of DNA histogram when P388/VCR-600 was treated with H-8. Thus the growth-inhibition by H-8 of P388 cells is mediated through the inhibition of cell cycle progression at $G_1$ and $G_2$ phase rather than through a killing of cells.

**Figure 2** The effect of H-8 **a**, and H-7 **b**, on the proliferation of P388 (○), P388/VCR (▲), and P388/VCR-600 (●). Cells were incubated with or without drugs for 48 h and viable cell number per well was counted. Points are mean±s.d. from triplicate determination of representative experiment. Three more experiments gave rise to the same results.

**Table II** The effect of H-8 on cell cycle

| | | Cell lines | | |
|---|---|---|---|---|
| | | P388 | P388/VCR | P388/VCR-600 |
| Control | $G_0/G_1$ (%) | 26.64 | 31.62 | 31.89 |
| | S (%) | 50.12 | 49.20 | 33.05 |
| | $G_2/M$ (%) | 23.22 | 19.16 | 33.04 |
| | CV (%) | 3.80 | 3.81 | 4.27 |
| Treatment with 60 µM H-8 | $G_0/G_1$ (%) | 40.40 | 34.47 | 32.32 |
| | S (%) | 25.41 | 40.58 | 39.61 |
| | $G_2/M$ (%) | 31.17 | 29.94 | 28.06 |
| | CV (%) | 7.40 | 5.11 | 4.04 |

P388, P388/VCR, and P388/VCR-600 cells were treated with 60 µM of H-8 for 48 h, washed twice with phosphate-buffered saline, fixed with ethanol, stained with propidium iodide, and analysed on Coulter EPICS-C flow cytometer.

**Table III** Distribution of A-kinase activity between cytosolic and membrane fractions

| Cell line | Number of experiment | A-kinase activity (pmol min$^{-1}$ mg$^{-1}$ protein)$^a$ Cytosol | Membrane | Total |
|---|---|---|---|---|
| P388 | 3 | 78.9±7.54 | 376.4±59.3 | 455.3±66.0 |
| P388/VCR-600 | 3 | 147.1±44.2 | 657.0±18.0 | 804.1±48.2 |
| P value$^b$ | | NS$^c$ | $P<0.025$ | $P<0.025$ |

$^a$Mean±s.d.; $^b$F-test; $^c$NS, not significantly different. A-kinase assay was done as described in Materials and methods and expressed as pmol $^{32}$P incorporated into Kemptide in 5 min at 30°C in the presence or absence of 10 µM cyclic AMP. In calculating A-kinase activity, cAMP-independent activity was subtracted from cAMP-dependent activity.

### *The effect of H-8 on the binding of $^3$H-vincristine to membrane vesicles*

Since P388/VCR and P388/VCR-600 overexpress P-glycoprotein which acts as a pump molecule and transports anticancer agents outside the cell, we initially predicted that P-glycoprotein should play an important role in the mechanism of cross-resistance to H-8. These predictions were tested by examining the effect of H-8 on the binding of $^3$H-vincristine to membrane fractions isolated from P388/VCR-600 cells. As shown in Figure 3, the binding of $^3$H-vincristine to membrane fraction is inhibited in a dose-dependent manner by vincristine, adriamycin, or verapamil whereas H-8 had no effect at the concentration up to 100 µM.

### *A-kinase and G-kinase activities*

To determine if the cross-resistance to H-8 observed in P388/VCR-600 cell is due to an increase in A-kinase or G-kinase activity, the enzyme activity of cytosol and membrane fraction from P388 and P388/VCR-600 cell lines was measured. Although A-kinase activity significantly increased in P388/VCR-600 cells (Table III), H-8 inhibited A-kinase activity of P388 and P388/VCR-600 cells in a similar rate (Figure 4). There was no detectable G-kinase activity in these cell lines.

**Figure 4** An inhibitory effect of H-8 on cyclic AMP-dependent phosphorylation of Kemptide by cytosol (□) and membrane (▩) fraction of P388 **a**, and those of P388/VCR-600 cells **b**. Concentrations causing 50% inhibition of A-kinase activities from P388 cytosol, P388 membrane, P388/VCR-600 cytosol, and P388/VCR-600 membrane are 3.37 µM, 3.46 µM, 2.19 µM and 2.81 µM respectively. Mean±s.d. of the triplicate determinations from a representative experiment. One more experiment showed same results. An absence of bar indicates the s.d. within the symbol.

### Discussion

Our data indicate that the growth of P388 cells, P388/VCR and P388/VCR-600 is uniformly inhibited by H-7 and there is no difference in the sensitivity to H-7 among these cell lines. However there is a significant difference in the sensitivity of cell lines to H-8. The growth of parental P388 is dose-dependently inhibited by H-8. However MDR cell lines showed resistance to H-8 according to their extent of resistance to vincristine, adriamycin, and VP-16. One possible explanation for the cross-resistance to H-8 might be related to an overexpression of P-glycoprotein and the increased efflux of H-8. If this were the case for cross-resistance to H-8, then P388/VCR and P388/VCR-600 would also show cross-resistance to H-7, because H-7 and H-8 belong to isoquinolinesulfonamide derivatives and have quite similar structures (Hidaka *et al.*, 1984). However we could not see any difference in the growth-inhibitory effect of H-7 among these cell lines. Furthermore, H-8 had no effect to inhibit the binding of $^3$H-vincristine to the membrane fractions isolated from P388/VCR-600 cells. Moreover, H-8 neither enhanced the accumulation of $^3$H-vincristine in P388/VCR-600 cells as examined by whole cell assay (data not shown), nor increased the sensitivity of P388/VCR-600 cells to vincristine and daunomycin (data not shown). These data suggest that the cross-resistance to H-8 observed in MDR P388 cell lines may be most probably mediated through a mechanism different from an overexpression of P-glycoprotein.

**Figure 3** Inhibition of $^3$H-vincristine binding to plasma membrane fractions isolated from P388/VCR-600 cells by H-8 (●), adriamycin (□), vincristine (O), or verapamil (Δ). Mean±s.d. of the triplicate determinations from a representative experiment. One more experiment showed same results. An absence of bar indicates the s.d. within the symbol.

Because the degree of resistance to H-8 is directly proportional to both the intensity of resistance to anticancer drugs such as vincristine, adriamycin, and VP-16 and the extent of P-glycoprotein expression, the mechanism of resistance to H-8 might be related to an amplification of a gene, the overexpression of which might be the result of its co-amplification with *mdr* (P-glycoprotein) gene.

H-7 and H-8 inhibit several protein kinases in a different extent. H-8 is reported to be the most potent inhibitor of cyclic nucleotide-dependent protein kinases and inhibits protein kinase C more weakly than H-7 and vice versa. We measured these kinase activities in these cell lines. Although A-kinase activity was about 1.77-fold higher in P388/VCR-600 cells than that in P388 cells, H-8 inhibited A-kinase activity or both cell lines in a similar rate. No detectable G-kinase activity was found in these cell lines. Therefore cross-resistance to H-8 may not be related to a change in A-kinase or G-kinase activity. Another explanation of this phenomenon is that H-8 may affect one or more targets unrelated to these protein kinases which could control regulatory pathways critical for the proliferation of the parental P388 cells but not for that of P388/VCR-600 cells.

From the data present here, it is tentatively concluded that the cross-resistance to H-8 observed in MDR variants of P388 cells is not due to an overexpression of P-glycoprotein or a change in A-kinase or G-kinase activity. Further studies are necessary to identify the cause of the resistance to H-8 in MDR cell lines.

We thank Dr V. Ling for providing us C219 monoclonal antibody. This work was supported in part by a Grant-in-Aid for scientific research for ministry of Education, Science and Culture of Japan.

### References

BATIST, G., TULPULE, A., SINHA, B.K. & 3 others (1986). Overexpression of a novel anionic glutathione transferase in multidrug-resistant human breast cancer cells. *J. Biol. Chem.*, **261**, 15544.

BECK, W.T., MUELLER, T.J. & TANZER, L.R. (1979). Altered surface membrane glycoproteins in vinca alkaloid-resistant human leukemic lymphoblasts. *Cancer Res.*, **39**, 2070.

BIEDLER, J.L., RIEHM, H., PETERSON, R.H.F. & SPENGLER, B.A. (1975). Membrane-mediated drug resistance and phenotypic reversion to normal growth behaviour of Chinese hamster cells. *J. Natl Cancer Inst.*, **55**, 671.

CHEN, C., CHIN, J.E., UEDA, K. & 4 others (1986). Internal duplication and homology with bacterial transport proteins in the mdr1 (P-glycoprotein) gene from multidrug-resistant human cells. *Cell*, **47**, 381.

CORNWELL, M.M., SAFA, A.R., FELSTED, R.L., GOTTESMAN, M.M. & PASTAN, I. (1986). Membrane vesicles from multidrug-resistant human cancer cells contain a specific 150- to 170 kDa protein detected by photoaffinity labeling. *Proc. Natl Acad. Sci. USA*, **83**, 3847.

DEFFIE, A.M., ALAM, T., SENEVIRATNE, C. & 5 others (1988). Multifactorial resistance to adriamycin: relationship of DNA repair, glutathione transferase activity, drug efflux, and P-glycoprotein in cloned cell lines of adriamycin-sensitive and -resistant P388 leukemia. *Cancer Res.,*, **48**, 3595.

GERLACH, J.H., ENDICOTT, J.A., JURANKA, P.F. & 4 others (1986). Homology between P-glycoprotein and a bacterial haemolysin transport protein suggesting a model for multidrug resistance. *Nature*, **324**, 485.

GROS, P., CROOP, J. & HOUSMAN, D. (1986). Mammalian multidrug resistance gene: complete cDNA sequence indicates strong homology to bacterial transport proteins. *Cell*, **47**, 371.

HAMADA, H. & TSURUO, T. (1988). Purification of the 170- to 180-kilodalton membrane glycoprotein associated with multidrug resistance. *J. Biol. Chem.*, **263**, 1454.

HIDAKA, H., INAGAKI, M., KAWAMOTO, S. & SASAKI, Y. (1984). Isoquinolinesulfonamides, novel and potent inhibitors of cyclic nucleotide dependent protein kinase and protein kinase C. *Biochemistry*, **23**, 5036.

HIGASHIGAWA, M., IDO, M., OHKUBO, T. & 5 others (1989). Increased sensitivity to 1-β-D-arabinofuranosylcytosine in P388 murine leukemic cells resistant to etoposide. *Leukemia Res.*, **13**, 39.

IDO, M., ASAO, T., SAKURAI, M. & 3 others (1986). 1-(5-Isoquinolinesulfonyl)-2-methylpiperazine (H-7) inhibits TPA-induced reduction of vincristine uptake from P388 murine leukemic cell. *Leukemia Res.*, **10**, 1063.

IDO, M., SATO, K., SAKURAI, M. & 4 others (1987). Decreased phorbol ester receptor and protein kinase C in P388 murine leukemic cells resistant to etoposide. *Cancer Res.*, **47**, 3460.

INABA, M. & JOHNSON, R.K. (1977). Decreased retention of actinomycin D as the basis for cross-resistance in anthracycline-resistant subline of P388 leukemia. *Cancer Res.*, **37**, 4629.

INAGAKI, M., WATANABE, M. & HIDAKA, H. (1985). N-(2-aminoethyl)-5-isoquinolinesulfonamide, a newly synthesized protein kinase inhibitor, functions as a ligand in affinity chromatography. *J. Biol. Chem.*, **260**, 2922.

JULIANO, R.L. & LING, V. (1976). A surface glycoprotein modulating drug permeability in Chinese hamster ovary cell mutants. *Biochim. Biophys. Acta.*, **455**, 152.

LAEMMLI, U.K. (1970). Cleavage of structural proteins during the assembly of the head of bacteriophage T4. *Nature*, **227**, 680.

MALLER, J.L., KEMP, B.E. & KREBS, E.G. (1978). *In vivo* phosphorylation of a synthetic peptide substrate of cyclic AMP-dependent protein kinase. *Proc. Natl Acad. Sci. USA*, **75**, 248.

MEYERS, M.B., MERLUZZI, V.J., SPENGLER, B.A. & BIEDLER, J.L. (1986). Epidermal growth factor receptor is increased in multidrug-resistant Chinese hamster and mouse tumor cells. *Proc. Natl Acad. Sci. USA*, **83**, 5521.

MIRSKI, S.E.L., GERLACH, J.H. & COLE, S.P.C. (1987). Multidrug resistant in human small lung cancer cell line selected in adriamycin. *Cancer Res.*, **47**, 25944.

NAITO, M., HAMADA, H. & TSURUO, T. (1988). ATP/$Mg^{2+}$ dependent binding of vincristine to the plasma membrane of multidrug-resistant K562 cells. *J. Biol. Chem.*, **263**, 11887.

ROSKOSKI, R. Jr. (1983). Assay of protein kinase. In *Methods of Enzymology*, Corbin, J.D. & Hardman, J.G. (eds). Vol. 99, pp. 3–6, Academic Press: New York.

SAFA, A.R., GLOVER, C.J., MEYERS, M.B., BIEDLER, J.L. & FELSTED, R.L. (1986). Vinblastine photoaffinity labeling of a high molecular weight surface membrane glycoprotein specific for multidrug-resistant cells. *J. Biol. Chem.*, **261**, 6137.

TOWBIN, H., STAEHELIN, T. & GORDON, J. (1979). Electrophoretic transfer of proteins from polyacrylamide gels to nitrocellulose sheets: procedure and some applications. *Proc. Natl Acad. Sci. USA*, **76**, 4350.

TSURUO, T., IIDA, H., TSUKAGOSHI, S. & SAKURAI, Y. (1982). Increased accumulation of vincristine and adriamycin in drug-resistant P388 tumor cells following incubation with calcium antagonists and calmodulin inhibitors. *Cancer Res.*, **42**, 4730.

VICKERS, P.J., DICKSON, R.B., SHOEMAKER, R. & COWAN, K.H. (1988). A multidrug-resistant MCF-7 human breast cancer cell line which exhibits cross-resistance to antiestrogens and hormone-independent tumor growth *in vivo*. *Mol. Endocrinol.*, **2**, 886.

ZUCKIER, G. & TRITTON, T.R. (1983). Adiamycin causes up regulation of epidermal growth factor receptors in actively growing cells. *Exp. Cell Res.*, **148**, 155.